# New Appliances and Things Medical

**Published:** 1907-03-02

**Authors:** 


### NEW APPLIANCES AND THINGS MEDICAL.

[We shall be glad to receive, at our Office, 28 & 29 Southampton Street, Strand, London, W.C., from the manufacturers, specimens of all new preparations and appliances which may be brought out from time to time.]

### SCOTT'S EMULSION OF PURE COD-LIVER OIL WITH HYPOPHOSPHATES OF LIME AND SODA.

(Scott and Bowne, Ltd., Manufacturing Chemists, London.)

Messrs. Scott and Bowne, have sent us a bottle of this well-known preparation. The value of cod-liver oil as a nutrient in wasting diseases of all kinds is perhaps one of the most firmly established of all the dietetic facts derived from clinical experience. The reason why cod-liver oil should be so superior to other fats of almost identical composition is by no means explained, but according to some authorities depends upon the fact that it contains traces of certain basic principles, among which we may mention jecorine. The preparation before us consists of an emulsion. An emulsion of an oil as compared with the oil itself has two advantages, the first is, it has a pleasanter taste; and the second, that it is slightly more digestible. All fats before absorption must be emulsified, and therefore an emulsion is to some extent a pre-digested food. Scott's emulsion, in addition to cod-liver oil, contains the hypophosphites of lime and soda. There is abundant clinical experience with regard to the value of these drugs in conditions of malnutrition, and their addition to cod-liver oil is certainly to be recommended upon therapeutical grounds. Scott's emulsion has a pleasant taste, and is, as a rule, exceedingly well borne by patients.

